# A Life-Threatening Metastasis of Renal Cell Carcinoma: An Endotracheal Metastasis

**DOI:** 10.7759/cureus.103806

**Published:** 2026-02-17

**Authors:** Günel Ahmadova, Irmak Akarsu, Aysegul Kurtoglu, Ali Celik, Muhammet Sayan

**Affiliations:** 1 Department of Thoracic Surgery, Faculty of Medicine, Gazi University, Ankara, TUR; 2 Division of Thoracic Surgery, Bartin State Hospital, Bartin, TUR

**Keywords:** bronchoscopy, endotracheal metastasis, jet ventilation, renal cell carcinoma, rijit bronchoscopy

## Abstract

Renal cell carcinoma is the most common malignancy of the urinary system, and nearly half of patients develop lung metastases at the time of diagnosis or during follow-up. Endotracheal metastases from renal cell carcinoma are exceedingly rare but may be life-threatening due to acute airway obstruction. In such cases, therapeutic interventions are primarily directed toward maintaining airway patency rather than achieving oncological control. Here, we report a case of endotracheal metastasis successfully excised using rigid bronchoscopy under general anesthesia with jet ventilation in a patient who had undergone nephrectomy for renal cell carcinoma three years earlier. The patient remained recurrence-free in the trachea at one-year follow-up.

## Introduction

Renal cell carcinoma (RCC) is the most common malignancy of the urinary system, accounting for approximately 2-3% of all cancers. The lungs are the usual site of metastasis, occurring in 30-50% of patients [1]. In contrast, endotracheal and endobronchial metastases are extremely rare and have been reported only as case reports in the literature. Therefore, a precise incidence rate for endotracheal metastasis of RCC has not been established. In cases of endotracheal involvement, the risk of fatal airway obstruction outweighs oncological expectations in determining the treatment strategy [2]. Herein, we present a case of an endotracheal metastatic lesion that was excised using rigid bronchoscopy under general anesthesia with jet ventilation in a patient who had undergone nephrectomy for RCC three years earlier. We preferred urgent bronchoscopic excision because the patient had near-complete upper airway obstruction.

## Case presentation

**Figure 1:**
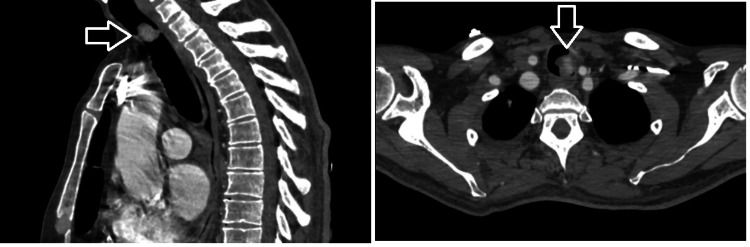
Preoperative thorax computed tomography of patient Sagittal and axial thoracic computed tomography scans demonstrating a mass lesion (arrow) in the upper trachea.

A 61-year-old man presented to the emergency department with progressive shortness of breath, stridor, and hemoptysis. His medical history included a right nephrectomy performed three years earlier for RCC, followed by immunotherapy for multiple pulmonary, hepatic, and bone metastases. On admission, the patient was tachypneic and orthopneic, with prominent inspiratory stridor. Oxygen saturation measured by pulse oximetry was 88% despite supplemental oxygen delivered via a simple face mask at 10 L/minute (medical oxygen source concentration ~100%, FiO₂ ~30-65%). Arterial blood gas analysis revealed hypoxemia, hypocapnia, and respiratory alkalosis. Thoracic computed tomography (CT) demonstrated a mass lesion with 20 × 25 mm radiological diameter at the tracheal inlet, causing near-complete airway obstruction (Figure 1).

**Figure 2:**
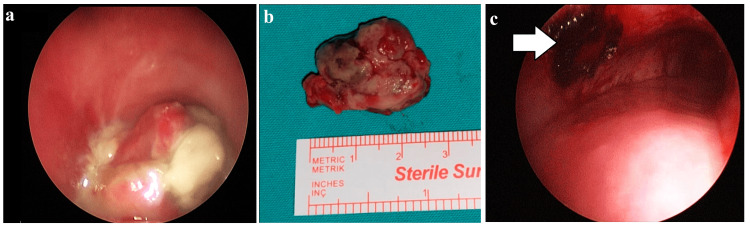
Bronchoscopic and macroscopic view of the endotracheal mass (a) Bronchoscopic view of a mass lesion almost completely obstructing the tracheal lumen. (b) Macroscopic appearance of the excised mass measuring approximately 2.5 cm in diameter. (c) Bronchoscopic view after excision demonstrating a patent tracheal lumen and the cauterized pedicle of the lesion (arrow).

The patient was urgently transferred to the operating room with a plan for rigid bronchoscopic excision. Following induction of general anesthesia, a jet ventilation catheter was advanced distal to the mass. Because the upper airway was completely obstructed, placement of a laryngeal mask airway, endotracheal intubation, or rigid bronchoscopy-guided intubation was not feasible for airway management. Although tracheotomy was a potential alternative, it was considerably more invasive than jet ventilation. A 12-mm rigid bronchoscope was passed through the vocal cords, revealing a pedunculated mass at the subcricoid level of the upper trachea that almost completely occluded the lumen (Figure 2a). The lesion was excised at its pedicle using an insulated cautery suction device. The tumor pedicle was cauterized and fully detached from the tracheal wall and subsequently removed with rigid forceps (Figure 2b-c). 

**Figure 3:**
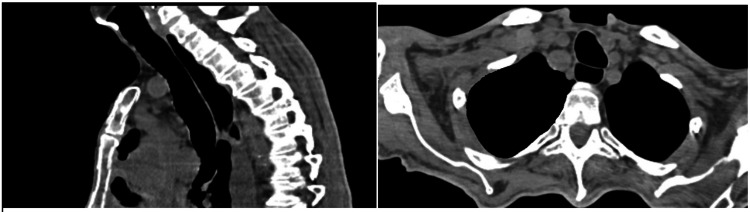
Patient's thoracic computed tomography scans taken one year postoperatively Thoracic computed tomography obtained one year after tracheal mass excision showing a patent tracheal lumen.

During the intraoperative period, no hypoxemia or hypercapnia was observed under jet ventilation support. Following the procedure, the patient was extubated and transferred to the intensive care unit. The postoperative course was uneventful, with stable vital signs. The patient was subsequently transferred to the ward without complications and discharged on postoperative day 2. Histopathological examination of the resected tracheal mass confirmed metastatic renal cell carcinoma. Macroscopic examination revealed an excised endotracheal mass measuring 2.6 × 2.5 × 1 cm. The specimen was off-white, with dark purple-black discoloration and a soft consistency. On sectioning, the cut surface appeared off-white to brownish and heterogeneous. Squamous metaplasia was identified in the tracheal epithelium at the base of the lesion. Immunohistochemical analysis revealed diffuse nuclear positivity for PAX-8 and diffuse cytoplasmic positivity for RCC, while p40 was negative in metastatic tumor cells. Based on the histopathological findings, the patient was re-referred to the medical oncology department during outpatient follow-up, and systemic therapy was modified to cabozantinib, a vascular endothelial growth factor receptor-tyrosine kinase inhibitor. Due to the presence of multiple metastases, local treatment was not planned. The tracheal metastasis was followed radiologically; because no recurrence was detected, radiotherapy was not planned. During one-year radiological follow-up, the tracheal lumen remained patent with no evidence of recurrence, and the patient experienced no further obstructive respiratory symptoms (Figure 3).

## Discussion

This case illustrates an exceptionally rare manifestation of RCC in the form of endotracheal metastasis. Although pulmonary metastases are common in RCC, respiratory symptoms vary depending on the number and location of metastatic lesions, lung parenchymal involvement, and the adverse effects of systemic therapies such as immunotherapy [3]. Endobronchial involvement in RCC may occur via lymphohematogenous spread or direct invasion [4]. In the present case, the absence of significant paratracheal lymphadenopathy, the distance between the pulmonary metastases and the trachea, and the pedunculated morphology of the lesion suggested hematogenous dissemination.

Clinical manifestations of endotracheal metastases typically include cough, dyspnea, stridor, and, less frequently, hemoptysis [5]. Our patient presented with a similar symptom profile, consistent with previous reports. Treatment selection for endotracheal metastases depends on several factors, including the location of the primary tumor, the number and distribution of metastatic lesions, and the longitudinal extent of tracheal involvement. Available treatment options include bronchoscopic interventions, tracheal resection, radiotherapy, and airway stent placement [6]. In this case, emergency bronchoscopic excision was chosen because of acute airway obstruction. Given the near-complete obstruction at the tracheal inlet, the procedure was performed under jet ventilation to ensure adequate ventilation of the distal airways. The pedicle base was cauterized to reduce the risk of local recurrence. As no recurrence was observed during the one-year follow-up, no additional interventional treatment for the tracheal metastasis was deemed necessary.

Securing the airway is critical during bronchoscopic excision of endotracheal masses. Airway management strategies range from laryngeal mask airway placement to extracorporeal membrane oxygenation in selected cases [7]. The use of jet ventilation in airway surgery has been reported as a standard approach in numerous studies, including randomized controlled trials, meta-analyses, and systematic reviews. The reported advantages of jet ventilation include improved surgical visibility and range of motion, uninterrupted ventilation, low fire risk with electrocautery, and prevention of airway damage [8]. In the present case, jet ventilation allowed safe bronchoscopic resection by ensuring adequate oxygenation distal to the lesion.

## Conclusions

Although endotracheal metastasis from RCC is extremely rare, it can occur and may present as an acutely life-threatening, near-complete airway obstruction. In such situations, urgent tumor resection via rigid bronchoscopy under jet ventilation should be strongly considered as a first-line strategy to secure the airway rather than to achieve oncological treatment.
